# Targeted memory reactivation during REM sleep may selectively enhance the late positive potential amplitude in previously encountered negative images: preliminary findings

**DOI:** 10.1093/sleepadvances/zpaf034

**Published:** 2025-05-24

**Authors:** Kazuki Sato, Satomi Okabe, Yoko Suzuki, Takashi Abe

**Affiliations:** Graduate School of Comprehensive Human Science, University of Tsukuba, Tsukuba, Ibaraki, Japan; International Institute for Integrative Sleep Medicine (WPI-IIIS), Tsukuba Institute for Advanced Research (TIAR), University of Tsukuba, Tsukuba, Ibaraki, Japan; Graduate School of Comprehensive Human Science, University of Tsukuba, Tsukuba, Ibaraki, Japan; International Institute for Integrative Sleep Medicine (WPI-IIIS), Tsukuba Institute for Advanced Research (TIAR), University of Tsukuba, Tsukuba, Ibaraki, Japan; Department of Applied Biological Chemistry, Graduate School of Agricultural and Life Sciences, The University of Tokyo, Bunkyo-ku, Tokyo, Japan; Japan Society for Promotion of Science, Chiyoda-ku, Tokyo, Japan; International Institute for Integrative Sleep Medicine (WPI-IIIS), Tsukuba Institute for Advanced Research (TIAR), University of Tsukuba, Tsukuba, Ibaraki, Japan; International Institute for Integrative Sleep Medicine (WPI-IIIS), Tsukuba Institute for Advanced Research (TIAR), University of Tsukuba, Tsukuba, Ibaraki, Japan

**Keywords:** REM sleep, emotion, late positive potential, targeted memory reactivation, olfactory stimulation

## Abstract

The function of rapid eye movement (REM) sleep in consolidating emotional memories and reducing emotional charge has been studied, but evidence remains conflicting. Our study employed the targeted memory reactivation (TMR) technique, which posits that specific sleep memories can be reactivated through sensory stimuli during sleep. Additionally, the late positive potential (LPP), a component of event-related brain potentials, was measured while participants (*N* = 16, 22.5 ± 1.2 years) viewed negative, neutral, or positive images (old images) paired with an odor stimulus. During subsequent REM sleep, the same odor was presented in the TMR condition, while an odorless stimulus was presented in the control condition. Upon awakening, participants performed the same task as before sleep, with new images added to test memory. The results demonstrated that TMR increased the LPP amplitude between 500 and 800 ms after image onset following sleep for negative old images; however, no changes were observed in the LPP in the same range for negative new images and neutral or positive images. TMR during REM sleep did not influence performance on the memory task, nor did it affect levels of arousal or emotional valence immediately after viewing the emotional images. These preliminary findings from our pilot study suggest that either the presentation of phenylethyl alcohol itself or the reprocessing induced by TMR during REM sleep selectively enhances the LPP in emotional processing of previously encountered negative stimuli. Due to the small sample size of this study, further investigation is warranted to evaluate the robustness of the results.

**Statement of Significance**

Previous studies investigating the hypothesis that REM sleep contributes to the consolidation of emotional memories and the attenuation of emotional responses have yielded conflicting results. This study investigated the effects of targeted memory reactivation (TMR) during REM sleep on emotional processing. During the experiment, odor stimuli paired with an emotional memory task involving unpleasant, pleasant, and neutral images, were re-presented during REM sleep. The results indicated no significant effects of TMR on emotional valence, arousal, or memory recall. However, TMR during REM sleep selectively enhanced neural responses to previously encountered negative stimuli after sleep. Replication and expanding these preliminary findings may contribute to a better understanding of the function of emotional processing during REM sleep.

## Introduction

Rapid eye movement (REM) sleep is characterized by activity in brain regions related to emotion, as evidenced by positron emission tomography [1, 2] and magnetic resonance imaging [3]. Additionally, magnetoencephalography [4] and electroencephalography [5] have indicated that the brain regions related to emotion become active prior to REMs during REM sleep. These findings demonstrate that REM sleep involves emotional processes, such as emotion depotentiation [6], emotional memory consolidation [7], and simulation of waking events to optimize behavior [8, 9].

The Sleep to Forget, Sleep to Remember hypothesis [6] indicates that the reactivation of emotional memories during REM sleep results in memory consolidation and a reduction in emotional charge. Some studies have demonstrated that sleep induces emotional charge reduction [10, 11], while others have shown no change [12, 13]. Therefore, conflicting evidence remains regarding this hypothesis, indicating the need for further research on the effects of REM sleep on emotion.

Since the importance of slow-wave sleep in declarative memory was first reported using the methodology of targeted memory reactivation (TMR) [14], TMR has been recognized as a key approach for investigating the role of sleep in memory consolidation [15]. TMR is believed to reactivate memories during sleep when sensory stimuli (such as olfactory or auditory stimuli) are paired with memory tasks before sleep [14–16]. It is assumed that this method allows for intervention in targeted memory content during sleep by using stimuli that were associated with the memory during wakefulness at precisely timed moments. This approach could minimize the influence of circadian rhythms or sleep deprivation. TMR has been shown to enhance both declarative and procedural memories [15, 17].

Some studies [18, 19] investigating the Sleep to Forget, Sleep to Remember hypothesis using TMR also showed controversial results. Lehmann et al. [19] demonstrated that TMR through auditory stimuli during Non-rapid eye movement (NREM) sleep enhances memory for emotional pictures but not during REM sleep nor during awake. In their study, participants first performed arousal ratings for neutral words before the learning phase and then engaged in an association learning task linking neutral words with emotionally arousing or non-arousing pictures. Retrieval sessions were conducted before and after TMR during NREM sleep, REM sleep, or wakefulness. After the post-sleep retrieval session, participants again rated the arousal levels of the neutral words. Additionally, they found no effect of TMR on subjective arousal rating on the neutral words which were paired with arousing or non-arousal pictures in either sleep state. Hutchison et al. [18] conducted an encoding task in which unpleasant or neutral images were presented together with associated sounds. Arousal rating tasks for the images were performed both before and after the encoding phase, and again after sleep. Half of the sound stimuli that were presented during encoding were replayed during either REM sleep or slow-wave sleep. They found that arousal on the next day was reduced for negative images paired with auditory stimuli presented compared to images paired with sounds that ware not presented during REM sleep. No such effect was observed during slow-wave sleep. Additionally, they observed no TMR effect on emotional memory performance, neither during slow-wave sleep nor REM sleep. Since these studies relied on subjective measures of emotional arousal, further research is warranted to verify the results using objective measures.

Notably, during REM sleep, particularly in the phasic phase, the brain’s response threshold to auditory stimuli increases [20–22], indicating that auditory stimulation for TMR may be less effective during this stage. In contrast, olfactory stimuli have been reported to trigger distinct reactions to pleasant and unpleasant odors [23] and even influence dream content during REM sleep [24]. Therefore, olfactory stimuli may be more suitable for TMR during REM sleep.

In studies where both memory and emotional reactivity have been measured simultaneously to investigate the role of REM sleep, emotional reactivity has been evaluated using cortisol levels [7] or event-related potentials [13]. Event-related potentials offer direct recordings of brain activity, providing high temporal resolution and quick responses to stimuli, making them more advantageous than endocrine indicators for objective assessment in emotional regulation. In studies investigating emotional regulation using event-related potentials, the late positive potential (LPP) is frequently assessed as a prominent measure [25], which is obtained by averaging electroencephalogram recordings time-locked to the onset of the image stimulus. The LPP amplitude elicited by emotional stimuli, both for negative and positive stimuli, is greater than that observed in neutral stimuli, which commences 300–400 ms after the presentation of the emotional stimulus and persists throughout and after the stimulus presentation [25–27]. The LPP comprises at least two subcomponents. Initially, it is dominant in the centroparietal region, but later, it becomes more prominent in the frontal region [25, 28]. The first subcomponent emerges in the early timeframe of 300–600 ms and shares similarities with the P3 component. The P3 component is typically observed over parietal regions and peaks around 300–400 ms. It is thought to reflect attentional allocation, activation of immediate memory, or arousal level [29]. The second subcomponent of the LPP appears later, beyond 600 ms, and is associated with one or more processes related to emotional processing [28]. Groch et al. [13] demonstrated that memory consolidation for emotional imagery was more pronounced than for neutral images during REM-rich sleep. The study further dissected the LPP, dividing it into early (300–500 ms post-stimulus) and late (500–800 ms post-stimulus) components. The results revealed that the early LPP in the frontal region for negative images exhibited a more pronounced increase following REM-rich late sleep than slow-wave sleep-rich early sleep. No such effect was observed for neutral images. The study could not completely rule out the potential effects of circadian rhythms because of the varying timing of tasks undertaken during REM-rich sleep and slow-wave sleep-rich sleep conditions. Hence, by facilitating the emotional memory reactivation through TMR during REM sleep, modulation of the amplitude of the LPP in response to emotional images could be observed without the potential effects of circadian rhythms.

This study aimed to investigate the effects of TMR with olfactory stimuli during REM sleep on memory consolidation and LPP for emotional images to elucidate the role of REM sleep in promoting emotional memory consolidation and emotional response reduction. We hypothesized that TMR during REM sleep reduces the LPP amplitude for emotional stimuli, which indicates reduced emotional response. Conversely, we hypothesized that TMR during REM sleep increases memory accuracy for emotional stimuli.

## Methods

### Participants

Thirty-six participants underwent the screening process, of which twenty-one were deemed eligible. Three declined to participate in the experiment after the adaptation night, and two declined after the first day of the experimental condition. Thus, this study analyzed data from the remaining 16 participants (age range: 20–25 years, mean ± standard deviation [SD]: 22.5±1.2 years). The characteristics of the participants are shown in Table 1. The participants had no current or prior history of sleep, psychiatric, olfactory, or taste disorders and met the requirements of questionnaires, including the Japanese version of the Pittsburgh Sleep Quality Index [30, 31] (≤5) and the Morning–Evening Questionnaire [32, 33] (30–70). The participants’ body mass index was within the range of 18.5 to 25.0 kg/m^2^. These participants led a regular lifestyle, with bedtimes from 09:00pm to 01:00am, waking times from 06:00am to 09:00am, and sleeping for 7 to 9 hours daily. They did not work night shifts after 10:00pm in the three months preceding the study, had not recently traveled to a country with a time difference greater than 3 hours compared to Japan, and were not on daily medication. The study excluded participants who were habitual alcohol drinkers (approximately > 40 g of alcohol at least twice a week), current smokers, or had a daily caffeine intake of > 400 mg.

**Table 1.** Characteristics of the participants (*N* = 16)

Variable	
Age (means ± SD), years	22.5 ± 1.2
Sex, *n*	
Men	10
Women	6
Body mass index (means ± SD), kg/m^2^	21.1 ± 1.6
Morningness–Eveningness questionnaire (means ± SD), score	51.6 ± 5.7
Morningness–Eveningness questionnaire –type, n	
Moderate Morning	1
Intermediate	15
Moderate Evening	0
Pittsburgh Sleep Quality Index (means ± SD), score	3.2 ± 1.0

Values are means ± SD.

Participants were evenly divided into two groups. One group completed the odor condition first, followed by the control condition, while the other group completed the control condition first, followed by the odor condition. The order of conditions was randomly assigned for each participant.

The study was approved by the University of Tsukuba Human Research Ethics Committee (2021-228A). It was conducted after obtaining written informed consent from all participants, who were apprized of the study’s purpose, methodology, and potential risks. This research was conducted in accordance with relevant guidelines and regulations, as well as the Declaration of Helsinki.

### Design and procedure

**Figure 1.**
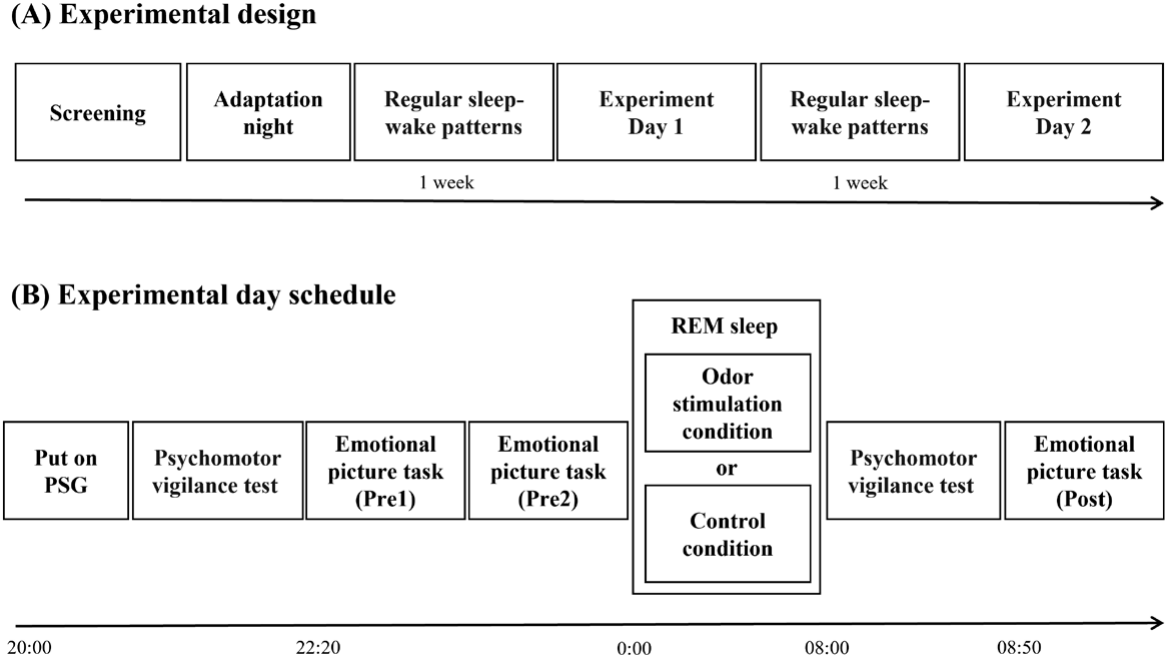
Experimental design and experimental day schedule. (A) After the participants had passed the screening online, an adaptation night was conducted for participants who had slept in the laboratory for the first time. After the adaptation night, the participants underwent a 1-week lifestyle control period to adjust their sleep–wake rhythms. Each participant spent one night in the experimental condition and another in the control condition. (B) Participants arrived at the laboratory at 20:00, where they applied the electroencephalogram electrodes. After undergoing the psychomotor vigilance test and completing a questionnaire, the emotional picture task was performed twice (pre-sleep 1 and pre-sleep 2) starting at 22:20. The participants went to bed at 24:00 and woke up at 08:00 the next day. When the experimenter detected REM sleep, odor or odorless stimuli were presented. After the participants woke up at 8:00 the next day, participants underwent psychomotor vigilance test at 08:30 and completed the questionnaire. Participants performed the emotional picture task (Post) starting at 8:50; electroencephalogram was recorded.

The study had a randomized, open-label, and crossover design. The experimental schedule is depicted in Figure 1A. Participants underwent an adaptation night in the laboratory if they were sleeping there for the first time. Before experiment day 1, participants were asked to adhere to a regular sleep–wake rhythm for 1 week. During this period, activities such as staying up all night, taking long naps, or excessive alcohol consumption were prohibited. Sleep indices for the week were obtained using MotionWatch 8 (CamNtech Ltd, Cambridgeshire, United Kingdom) to ensure that participants adhered to the sleep–wake cycle prior to each experimental day.

The schedule for the experimental day is illustrated in Figure 1B. Participants spent one night each for the experimental condition (odor condition) and control condition. The same participants were exposed to odor and control conditions. Participants were asked to refrain from napping, excessive exercise, and alcohol consumption the day before the experiment. They were also instructed to abstain from smoking and taking caffeine on each experiment day. After participants arrived at the laboratory at 10:00pm, electrodes for polysomnography were attached. They subsequently underwent a 10-minute psychomotor vigilance test [34, 35] and completed the Japanese version of the Karolinska sleepiness scale [36, 37] and profile of the mood states second edition-adult short questionnaires [38, 39]. At 10:20pm, participants performed the emotional picture task. They went to bed at 12:00am and awoke at 08:00am the next day. During the night, the experimenter monitored the participant’s polysomnogram and presented olfactory (odor condition) or odorless stimuli (control condition) during REM sleep. Upon awakening, the participants spent 30 minutes in the laboratory to reduce the effect of sleep inertia. They underwent a 10-minute psychomotor vigilance test starting at 08:30am, completing the Karolinska sleepiness scale and profile of the mood states 2. The emotional picture task was performed from 08:50am. After all procedures were completed on the final day of the experiment, participants received additional information about which nights the olfactory or odorless stimuli were delivered during sleep.

### Emotional picture task

This study used 840 (280 positives, 280 negatives, and 280 neutral) images obtained from the International Affective Picture System [40] and Open Affective Standardized Image Set [41], and created four sets of 210 images (70 positive, 70 negative, and 70 neutral) per set with equivalent emotional valence (negative: 2.39 ± 0.64, neutral: 5.17 ± 0.44, and positive: 6.85 ± 0.79) and arousal (negative: 5.77 ± 0.74, neutral: 3.31 ± 0.47, and positive: 5.24 ± 0.83) (Supplementary Table S1). Four sets of 210 images were used in this study with the same ratio (International Affective Picture System: 55 images; Open Affective Standardized Image Set: 15 images) for each image type. Before sleep, participants were presented with 210 images (old images), and subjective ratings of these images were obtained using a self-assessment manikin [42]. In addition, 210 new images (new images) were added to the task to test memory during the emotional picture task after waking. The Latin square method was used to assign the four sets to the odor-old, odor-new, control-old, and control-new images.

**Figure 2.**
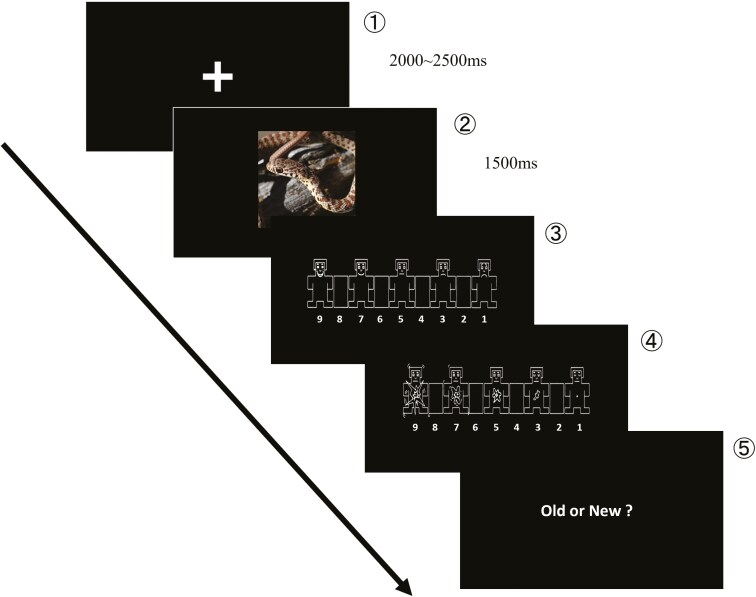
Emotional picture task. After cross fixation first appeared randomly (2.0–2.5 seconds) (1), negative, neutral, or positive images were presented (1.5 seconds) (2). Participants were required to rate their valence (3) and arousal (4) using a self-assessment manikin. At the post-task, participants completed a memory test in addition to the pre-sleep procedure. Participants were asked to discriminate whether the presented picture was old or new in this test (5). Images were retrieved from Openverse ("SNAKE COILED ON LOG - CROOKED RIVER NATIONAL GRASSLAND" by the Forest Service Pacific, Northwest Region, available under Public Domain Mark 1.0 at https://wordpress.org/openverse/image/43b00faa-f58b-4e3d-822b-cfb5ae28f269) and the Self-Assessment Manikin as described previously [42].

Participants were asked to rate their emotional valence and arousal in response to the emotional images (Figure 2). Visual stimuli were presented on a 14.1-inch personal computer and controlled by Psychopy 3 (ver. 2021. 1.4) [43]. The viewing distance from the display was 57 cm. In the task, the cross fixation (1.9 × 1.9°) first appeared randomly on a black background for 2.0 to 2.5 seconds. After that, emotional images (14.25 × 14.25°) selected from the International Affective Picture System or Open Affective Standardized Image Set were presented for 1.5 seconds. When the self-assessment manikin of emotional valence appeared on the screen, the participants rated the emotional valence from 1 to 9 (1 = negative, 5 = neutral, and 9 = positive). Finally, when the self-assessment manikin of arousal appeared on the screen, the participants rated their arousal (1 = calmness, 5 = normal, and 9 = arousal). This experiment used the same set of images twice before bedtime (pre-sleep 1 and pre-sleep 2) to fully connect with the olfactory and visual stimuli. In addition to the pre-sleep procedure, a memory test was conducted after waking up. Participants needed to answer whether they had seen the image before sleep (“old”) or not (“new”). Participants were instructed about the memory test after sleep at the screening session.

### Affective rating and memory performance

The mean rating of valence and arousal for each condition was calculated for each image type before and after sleep (pre-sleep 1 and pre-sleep 2, and old and new, respectively). This study also calculated the overnight habituation index by subtracting the post-arousal value for old images from the arousal value in pre-sleep 2 and dividing it by the arousal value in pre-sleep 1 according to the previous TMR research [18].

Memory performance after waking up was evaluated using the hit rate and *d’*. The hit rate was calculated as the percentage of responses correctly identifying that the old image had been seen before going to bed. Correct rejection was determined by the percentage of responses accurately answering that the new image had not been seen before sleep. The value of *d’* was subsequently obtained by subtracting the z-transformed 1-correct rejection from the z-transformed hit rate.

### Olfactory stimulation

Olfactory stimuli were presented before bedtime and after waking up during the emotional picture task using an olfactory stimulus presentation device [24]. The airflow, generated by a HIBLOWAIR PUMP CD-8 (Techno Takatsuki Corporation, Osaka, Japan), was regulated at a flow rate of 4 L/min through a flowmeter equipped with a precision needle valve (KOFLOC Corporation, Kyoto, Japan). The odor was delivered through bubbling in a reagent bottle using undiluted phenylethyl alcohol (Wako Pure Chemical Industries, Ltd., Osaka, Japan) as the olfactory stimulus, while odorless distilled water (high-purity purified water, Furukawa Chemical Industries, Ltd.) was used in the control condition. The stimuli were alternated on and off every 30 seconds to avoid habituation and preserve nasal hydration, as previously demonstrated [14]. The participant’s nasal tip was fitted with a tube for dispensing the odor.

The participants were verbally queried regarding their perception of the olfactory stimuli before each emotional task (before bedtime and after waking up) to confirm their sense of smell on the experiment day. Participants were subjected to an 8-hour sleep period during which olfactory stimuli were delivered during all the REM sleep stages. Olfactory stimuli were delivered upon detection of REM sleep. The detection of REM sleep was performed in real-time using the criteria of low amplitude of electroencephalogram, low muscle tone, and REMs according to the American Academy of Sleep Medicine Manual for Scoring of Sleep and Associated Events ver. 2.5 [44]. The stimuli were discontinued upon the participant’s arousal or transition into another sleep stage. The experiment was carefully monitored from outside the laboratory to ensure accurate delivery of the olfactory stimuli.

### EEG measurement and analysis

During the task and sleep, an electroencephalogram (Fpz, Fz, Cz, Pz, F3, F4, C3, C4, O1, O2, M1, and M2) was recorded using an electroencephalograph (Polymate Pro MP6100, Miyuki Giken, Tokyo, Japan) based on the international 10–20 method with FCz as a system reference. Electrooculogram electrodes were placed 1 cm lateral to the left and right outer canthi to monitor horizontal eye movements, and 1 cm above and below the left eye to monitor vertical eye movements. Electrodes were also placed to record two chin electromyograms. During the emotional picture task and sleep, electroencephalogram, electrooculogram, and electromyogram were recorded using a sampling frequency of 1000 Hz and a recording frequency range of direct current to 330 Hz.

Electroencephalogram, electrooculogram, and electromyogram were re-referenced and used to determine the sleep stages according to the American Academy of Sleep Medicine [44]. The sampling frequency was downsampled at 500 Hz for Electroencephalogram, electrooculogram, and electromyogram. For the electroencephalogram and electrooculogram, the low- and high-frequency filters were set at 0.3 and 35 Hz, respectively. The notch filter was 50 Hz. The electromyogram was filtered from 10 to 100 Hz. Sleep scoring (Stages W, N1, N2, N3, and REM) was performed by dividing 30-second epochs. The number of REMs during REM sleep was analyzed using an automatic eye movement detection program [45]. The automatically collected data identified some instances unrelated to REM. Thus, one of the authors (KS) visually inspected the detected REMs and excluded these events from the analysis. The frequency of REMs was calculated by dividing the number of REMs by the total duration of REM sleep.

EEGLAB (ver 2022.0) [46] in MATLAB (The Mathworks, Natick, MA) was used to analyze the Event-related potentials. Electroencephalogram and electrooculogram data during the task were pre-processed using EEGLAB by re-referencing the averaged amplitude of both mastoids (M1 and M2). First, low- and high-cut filters were applied at 1 and 40 Hz, respectively, to perform independent component analysis and eliminate blink and eye movement components. Then, the acquired independent component analysis information was applied to unfiltered, re-referenced electroencephalogram/electrooculogram data, which was filtered at 0.1–40 Hz and notched at 50 Hz. The epoch was set from 100 ms before to 1000 ms after the presentation of the emotional image stimulus, and the interval of 100 ms before the stimulus presentation was used for baseline correction. In addition, the trials with a potential of ± 80 μV or more during this interval were not included in the analysis. Following stimulus onset, the LPP amplitudes were measured for two specific time windows (300–500 ms and 500–800 ms). These amplitudes were obtained separately for positive, negative, and neutral images, both before bedtime and after waking up, as well as for the old and new images.

### Questionnaire

The Japanese version of the Karolinska sleepiness scale [36, 37] was used to assess the participants’ subjective sleepiness. Participants were asked to rate their sleepiness on a 9-point scale, with higher scores indicating greater sleepiness. The profile of the mood states 2 [38, 39] was used to assess the participants’ mood states. The profile of the mood states 2 consists of 35 questions on seven scales: anger-hostility, confusion-bewilderment, depression-dejection (DD), fatigue-inertia (FI), tension-anxiety (TA), vigor-activity, and friendliness. These scales were transformed into T scores. Total Mood Disturbance was calculated by dividing vigor-activity by the sum of anger-hostility, confusion-bewilderment, DD, FI, and TA. Higher T scores for anger-hostility, confusion-bewilderment, DD, FI, TA, and total mood disturbance indicated that the participants reported a stronger negative mood. Moreover, two positive mood states (vigor-activity and friendliness) and higher T scores indicated that participants reported a stronger positive mood.

### Statistical analyses

When normality was confirmed, a linear mixed model was employed. When normality was not confirmed in the raw data, BoxCox transformations were applied when the data were greater than 0. A Yeo–Johnson transformation was performed if the data contained numbers ≤ 0. Once normality was achieved, a linear mixed model was employed.

The LPP amplitudes, emotional arousal, memory performance, and questionnaire measures were analyzed individually using a linear mixed model. Statistical analyses of the LPP amplitudes and arousal during the pre-sleep task were conducted using the following fixed effect: condition (odor condition and control condition), image type (negative, neutral, positive), channel electrode (LPP amplitude only; Fpz, Fz, Cz, Pz, F3, F4, C3, C4, O1, and O2), and the interaction of these effects. The order of the conditions was used as a repeated effect, and each participant was selected as a block of the covariance structure in the linear mixed model. Statistical analyses of the LPP amplitude and arousal during the post-sleep task were conducted using linear mixed models with the condition, image type, channel electrode (for LPP amplitude only), image novelty (old and new), and their interactions as fixed effects, order of condition as repeated effects, and each participant as a block in the covariance structure. The statistical analyses of the memory accuracy items were analyzed separately using linear mixed models with the condition, image type, their interactions as fixed effects, and the order of the conditions as repeated effects. In this analysis, no criteria were established to ensure the validity of memory performance, and all the data were included. The mean reciprocal response time (1/response time [s]) in the psychomotor vigilance test was analyzed using a linear mixed model with fixed effects for condition, time (before and after sleep), and their interaction, and repeated effects for the order of conditions. Statistical analyses of the sleep variables and the average sleep duration 1 week before the experimental day obtained from MotionWatch8 were analyzed separately using a linear mixed model with the condition and their interaction as fixed effects and the order of the conditions as repeated effects. The covariance structures were applied from Auto Regression, Unstructured, Compound Symmetry, or Variance Components, following which the structure with the lowest Akaike’s Information Criterion was selected. For the linear mixed model, *PROC MIXED* (SAS Version 9.4, SAS Institute Inc, Cary, NC) was employed using the Kenward–Rogers method for the covariance parameters. The Bonferroni method was used for post-hoc comparison. The significance level was set at *p* < .05.

Since no normality was observed for the sleep variables (latency to fall asleep, N2 latency), questionnaires (Karolinska sleepiness scale, profile of the mood states: anger-hostility, confusion-bewilderment, DD, FI, and TA and total mood disturbance) and subjective ratings (valence), the Friedman test was performed using IBM SPSS Statistics Version 25.0 (Armonk, NY: IBM Corp.). The Friedman test was performed to test the differences among the four sessions (before and after sleep in odor and control conditions). When statistical significance was observed, the Wilcoxon signed-rank test with the Bonferroni correction was performed for post-hoc comparison. For the sleep variables, the Wilcoxon signed-rank test was used to confirm the presence of a difference between the two conditions.

To reduce the risk of Type 2 errors, additional analyses without multiple comparison corrections were conducted. In the original analyses, no outliers were excluded. To assess the robustness of the key indices obtained from the original analyses, the same analyses were repeated after excluding outliers. Outliers were defined as values smaller than the first quartile—1.5 times the interquartile range or larger than the third quartile + 1.5 times the interquartile range.

## Results

### Sleep variables

The average sleep duration for 1 week before each experimental day showed no significant difference between the two conditions (*F*(1, 30) = 0.00, *p* = .94). A linear mixed model demonstrated no significant difference in the presentation time of TMR stimulation during sleep between the conditions (*F*(1, 30) = 0.28, *p* = .60). The results for each sleep variable in the odor and control conditions are shown in Table 2. The results of the sleep variables using a linear mixed model and Wilcoxon signed-rank test demonstrated the absence of a main effect of condition on any of the variables, number of REMs, and the REM frequency (Table 2).

**Table 2.** The Result of the Sleep Variables

Sleep variable	Control	Odor	*F*(1,30)	*Z*	*P*
Total sleep time (min)	460.3 ± 9.2	460.8 ± 12.6	0.02		.90
Sleep efficiency (%)	95.9 ± 1.9	96.5 ± 2.6	0.22		.64
Sleep latency (min)	3.5 ± 2.8	3.6 ± 3.5		0.00	.00
Wake after sleep onset (min)	16.2 ± 9.3	15.6 ± 12.1	0.40		.53
N1 duration (min)	44.3 ± 21.1	39.0 ± 17.5	0.55		.46
N2 duration (min)	211.4 ± 27.7	228.8 ± 33.8	2.39		.13
N3 duration (min)	108.7 ± 27.7	99.5 ± 29.2	0.77		.39
REM duration (min)	96.0 ± 15.7	93.4 ± 26.7	0.10		.75
N2 latency (min)	7.2 ± 3.9	6.5 ± 4.3		−0.15	.65
N3 latency (min)	15.9 ± 4.8	15.9 ± 8.4	0.22		.65
REM latency (min)	103.0 ± 40.5	118.3 ± 50.6	0.83		.37
Number of REMs	543.3 ± 279.1	532.8 ± 284.6	0.01		.92
Frequency of REMs (number/min)	5.5 ± 2.4	5.5 ± 2.6	0.00		.98

Date are presented as mean ± SD. REM: rapid eye movement; *F*, *F* value for liner mixed model; *Z*, *Z* value for Wilcoxon signed-rank test; *p*, *p*-values for liner mixed model or Wilcoxon signed-rank test.

### Sleepiness and fatigue

**Figure 3.**
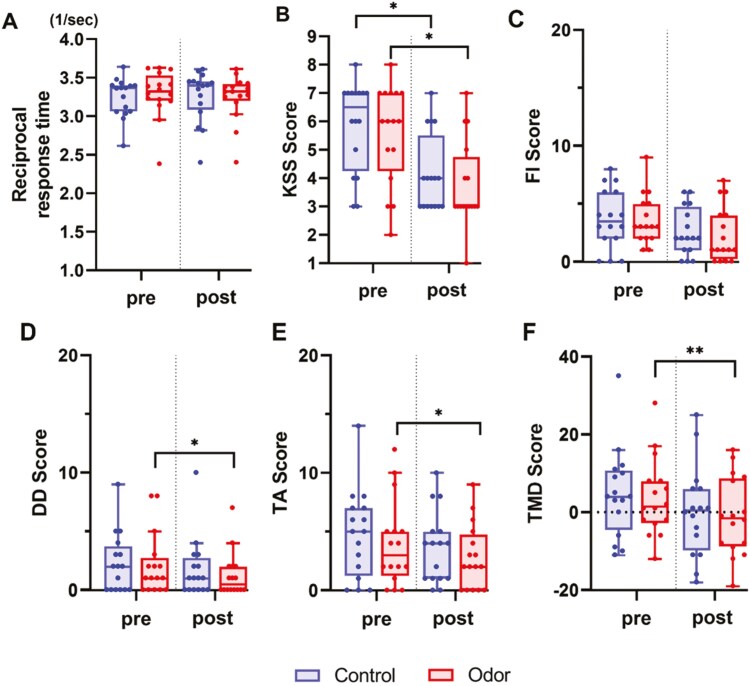
Sleepiness and mood before bedtime (Pre) and after waking (Post). (A) Mean reciprocal response time (1/s) in the psychomotor vigilance test, (B) Karolinska Sleepiness Scale, (C) FI in profile of mood states second edition-adult short. No significant differences existed between the odor and control conditions regarding sleepiness and fatigue before the emotional picture task. (D) DD. (E) TA. (F) Total Mood State in profile of mood states 2. The odor condition during REM sleep decreases next-day depression and the overall negative affect. Red boxes represent the odor condition, and blue boxes represent the control condition. Tukey plots illustrate the box plots, which show the median (central line), 25th percentile, and 75th percentile (lower and upper box limits). Tukey error bars indicate 1.5 times the 25th and 75th percentiles or maximum and minimum values. *:*p *< .05 with Bonferroni correction, **:*p *< .01 with Bonferroni correction

Figure 3A shows the pre-sleep and post-sleep results of the reciprocal response time of the psychomotor vigilance test conducted before the emotional picture task. No main effects of condition (odor vs. control condition), session (pre-sleep vs. post-sleep), or interaction between the condition and session were found for the reciprocal response time (condition: *F*(1, 60) = 0.08, *p* = .77; session: *F*(1, 60) = 0.01, *p* = .93; condition ⋅ session: *F*(1, 60) = 0.59, *p *= .45).

We tested the difference in subjective sleepiness between the conditions using the Karolinska sleepiness scale. The pre- and post-sleep results are shown in Figure 3B. The Friedman test (*df* = 3, *χ2* = 15.29, *p* = .002) and subsequent Wilcoxon signed-rank test revealed a difference between the pre-sleep and post-sleep sessions in both the conditions, with a decrease after sleep (odor condition: *Z* = −2.72, *p *= .024; control condition: *Z* = −2.51, *p* = .048, Bonferroni corrected). However, no significant differences were observed between the conditions in the sleepiness before and after sleep (before sleep: *Z *= −0.46, *p* = 1.00; after sleep: *Z* = −0.77, *p* = 1.00, Bonferroni corrected).

The Friedman test revealed differences in the four conditions (*df* = 3, *χ2* = 10.94, *p* = .012) for the FI of the profile of the mood states. However, the Wilcoxon signed-rank test revealed no significant differences between the conditions (before sleep: *Z* = −0.14, *p* = 1.00; after sleep: *Z* = −0.64, *p* = 1.00; Bonferroni corrected, Figure 3C) and before and after sleep (odor condition: *Z* = −2.42, *p* = .064; control condition *Z *= −1.80, *p* = .29; Bonferroni corrected, Figure 3C). When multiple comparison correction was not applied, the decrease in FI after sleep in the odor condition was statistically significant (odor condition: *p* = .016; control condition: *p* = .072).

### Mood

We examined the effects of olfactory stimulation during REM sleep on mood by analyzing each item of profile of the mood states 2 (Figure 3D–F). The Friedman test revealed the main effect of the four sessions for DD, TA, and total mood disturbance (DD: *df* = 3, *χ2* = 8.07, *p* = .045; TA: *df* = 3, *χ*^2^ = 7.98, *p* = .046; total mood disturbance: *df *= 3, *χ2* = 15.88, *p* = .001). Subsequent Wilcoxon signed-rank test showed a significant difference between the sessions in the odor condition for DD, TA, and total mood disturbance (DD: *Z* = −2.59, *p* = .040; TA: *Z* = −2.53, *p *= .044; total mood disturbance: *Z* = −3.06, *p* = 0.008; Bonferroni corrected, Figure 3D–F). In contrast, no significant differences were found between sessions in the control condition with Bonferroni correction (DD: *Z* = −1.73, *p* = .34; TA: *Z* = −1.74, *p* = .32; total mood disturbance: Z = −2.30, *p* = .084; Bonferroni corrected). When multiple comparison corrections were not applied, a significant difference in total mood disturbance between pre- and post-sleep was observed in the control condition (DD: *p* = .084; TA: *p *= .081; total mood disturbance: *p* = .021). Friedman tests for anger-hostility and confusion-bewilderment demonstrated no statistical significance (anger-hostility: *df* = 3, *χ*^2^ = 4.19, *p* = 0.24; confusion-bewilderment: *df* = 3, *χ2* = 5.62, *p* = .132) (Supplementary Figure S1A and B). The positive scales of vigor-activity and friendliness also found no main effect in the condition (vigor-activity: *F*(1, 60) = 1.01, *p* = 0.32, friendliness: *F*(1, 60) = 0.79, *p* = .38), session (vigor-activity: *F*(1, 60) = 0.05, *p* = 0.82, friendliness: *F*(1, 60) = 0.76, *p* = .387), or interaction between the condition and session (vigor-activity: *F*(1, 60) = 0.61, *p* = .44, friendliness: *F*(1, 60) = 0.09, *p* = .76) (Supplementary Figure S1C and D).

To examine the robustness of these results, analyses excluding outliers (DD: 5 data points, TA: 1 data point; total mood disturbance: 1 data point) were conducted. The Friedman test did not reveal statistically significant main effects of the four sessions for DD and TA (DD: *df* = 3, *χ2* = 7.09, *p* = 0.069; TA: *df* = 3, *χ*^2^ = 7.24, *p* = .065). For total mood disturbance, the result of the Friedman test was statistically significant (*df *= 3, *χ*^2^ = 14.80, *p* = .002). Wilcoxon signed-rank test showed a significant decrease after TMR in the odor condition for Total Mood Disturbance (*Z* = −3.056 *p* = .008; Bonferroni corrected), but not in the control condition (*Z *= −2.074, *p* = .152; Bonferroni corrected). When multiple comparison correction was not applied, total mood disturbance significantly decreased after sleep in both the odor and control conditions (odor condition: *p* = .002; control condition: *p* = .038).

### Subjective rating during the emotional picture task

**Figure 4.**
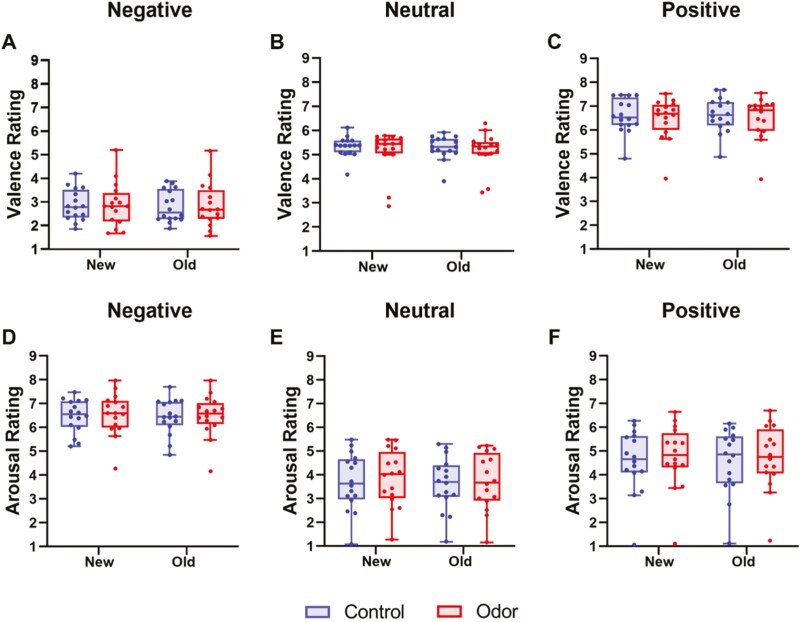
Affective rating after sleep. Valence (a–c) and arousal (d–f) of the new and old images. Negative (a, d), neutral (b, e), positive (c, f). No differences in the valence and arousal were found between the conditions after waking. Red boxes represent the odor condition, and blue boxes represent the control condition. Tukey error bars indicate 1.5 times the 25th and 75th percentiles or maximum and minimum values.

The emotional valence for each image type during the post-sleep session is shown in Figure 4A–C. Regarding valence, the Friedman test demonstrated no statistical significance for each image type (negative: *df* = 3, *χ2* = 0.63, *p* = .89, Figure 4A; neutral: *df* = 3, *χ2* = 1.9, *p* = 060, Figure 4B; positive: *df *= 3, *χ2* = 1.00, *p* = .80, Figure 4C) among four measurements (condition ⋅ novelty). The Friedman test excluding outliers (neutral images: 6 data points; positive images: 2 data points) also showed no statistical significance (neutral: *df* = 3, *χ2* = 0.67, *p* = .88; positive: *df *= 3, *χ2* = 0.54, *p* = .91). Considering valence before sleep (pre-sleep 1 and pre-sleep 2), the Wilcoxon signed-rank test revealed no significant differences between the two conditions for each image type (pre-sleep 1: negative: *Z* = −0.26, *p* = .80, neutral: *Z* = −0.98, *p* = .33, positive: *Z* = −0.49, *p* = .62; pre-sleep 2: negative: *Z* = −0.88, *p* = .38, neutral: *Z* = -0.28, *p* = .78, positive: *Z* = −0.62, *p* = .54).

The arousal results for each image type during the post-sleep session are shown in Figure 4D–F. A linear mixed model demonstrated a main effect of image type (*F*(2, 180) = 98.70, *p* < .0001). However, no effects of condition (*F*(1, 180) = 0.51, *p* = .47), image novelty (old vs. new; *F*(1, 180) = 0.14, *p* = .71), condition ⋅ image type (*F*(2, 180) = 0.03, *p* = .97), type ⋅ novelty (*F*(2, 180) = 0.03, *p* = .97), and condition ⋅ novelty interaction (*F*(2, 180) = 0.01, *p* = .90) were observed. The analysis excluding outliers (negative images: 2 data points; positive images: 4 data points) similarly showed only a main effect of type (*F*(2, 174) = 134.17, *p* < .0001). Considering the overnight habituation index [18], no main effect of the image type (*F*(2, 90) = 0.11, *p* = .90), the main effect of the condition (*F*(1, 90) = 0.20, *p* = .65), or condition ⋅ image type interaction (*F*(2, 90) = 0.60, *p* = .55) were observed. The analysis excluding outliers (negative images: 4 data points; positive images: 1 data point) similarly showed no statistically significant main effects or interactions. Arousal before sleep (pre-sleep 1 and pre-sleep 2) analyzed using a linear mixed model demonstrated a main effect of image type (pre-sleep 1: *F*(2, 90) = 43.33, *p* < .0001; pre-sleep 2: *F*(2, 90) = 50.38, *p* < .0001). Comparisons of the arousal of each image type in the emotional picture task before sleep (pre-sleep 1 and pre-sleep 2) confirmed that the arousal was higher for the emotional images than for the neutral images. Furthermore, arousal was higher for negative images compared to positive images. However, neither main effect of condition (pre-sleep 1: *F*(1, 90) = 0.00, *p* = .96; pre-sleep 2: *F*(1, 90) = 0.16, *p* = .69) nor condition ⋅ image type interaction (pre-sleep 1: *F*(2, 90) = 0.16, *p* = .85, pre-sleep 2: *F*(2, 90) = 0.04, *p* = .96) were found. In summary, no difference was observed in emotional valence and arousal between the odor and control conditions for both the pre- and post-sleep tasks.

### Event-related potential during the emotional picture task

**Figure 5.**
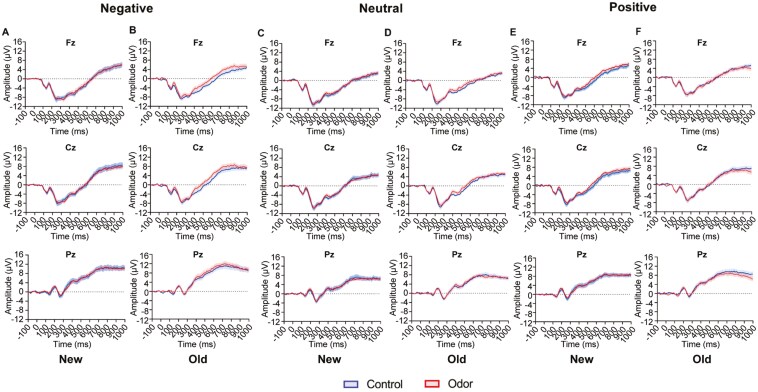
Averaged event-related potential (μV) recorded from Fz, Cz, Pz of the new (A, C, E) and old images (B, D, F) after waking up for negative (A, B), neutral (C, D), and positive (E, F) images. Red lines indicate the odor stimulation condition, and blue lines indicate the control condition. Shaded areas represent the standard error of the mean.

Figure 5 shows the event-related potentials during the emotional picture task for new and old image types after sleep at Fz, Cz, and Pz. Supplementary Figures S2–S4 present all 10 scalp electrodes and four EOG channels after sleep. The Yeo–Johnson transformations of all the LPP amplitudes (300–500 ms and 500–800 ms) confirmed normality; thus, a linear mixed model was used to analyze the data.

The main effect of image novelty was observed in the emotional picture task after sleep at 300–500 ms (*F*(1, 1800) = 49.51, *p* < .0001) and 500–800 ms (*F*(1, 1800) = 27.27, *p* < .0001), indicating that LPP amplitude increased for old images compared to new images in the post-sleep task. A main effect of the image type was also observed for the emotional picture task both before and after sleep at 300–500 ms (pre-sleep 1; *F*(1, 900) = 4.81, *p* = .01, Supplementary Table S2; pre-sleep 2: *F*(1, 900) = 3.69, *p* = .0025, Supplementary Table S3; Post: *F*(1, 1800) = 18.94, *p* < .0001, Supplementary Table S4) and 500–800 ms (pre-sleep 1: *F*(1, 900) = 38.91, *p* < .0001, Supplementary Table S2; pre-sleep 2: *F*(1, 900) = 25.23, *p* < .0001, Supplementary Table S3; Post: *F*(1, 1800) = 56.29, *p* < .0001, Supplementary Table S4). Multiple comparisons confirmed that the LPP amplitudes of negative and positive images were larger than those of the neutral images from 500 to 800 ms in all the sessions. The main effect of the electrodes at 500–800 ms was observed both before and after sleep (pre-sleep 1: *F*(1, 900) = 91.38, *p* < .0001; pre-sleep 2; *F*(1, 90) = 79.95, *p* < .0001; Post: *F*(1, 1800) = 160.44, *p* < .0001), with the largest amplitude at the parietal region, similar to a previous study [47].

At 300–500 ms, a main effect of condition was obtained for the emotional picture task after sleep (*F*(1, 1800) = 4.05, *p* = .044), with greater LPP amplitude in the odor condition (−1.54 ± 2.33 μV) than in the control condition (−1.87 ± 1.86 μV). No interaction involving the condition was observed (Supplementary Table S4). After excluding outliers for each combination of Type, Novelty, and electrode (13 data points), the linear mixed model indicated that the final Hessian matrix was not positive definite. Therefore, the model was simplified by removing the interaction from the fixed effect, leaving only the main effects. As a result, the main effect of the condition was statistically significant and showed a similar trend (*F*(1, 1800) = 4.69, *p* = .0304).

**Figure 6.**
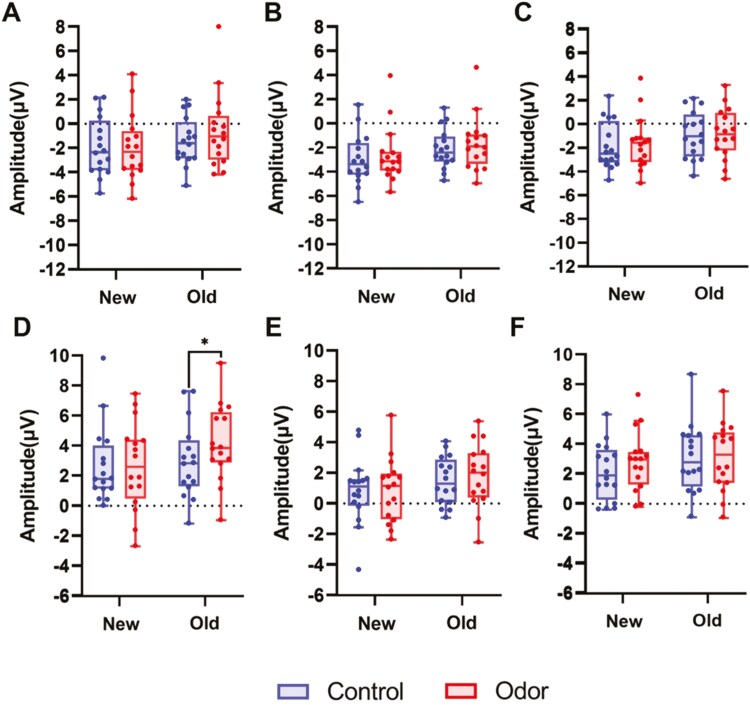
Late positive potential (LPP) amplitudes averaged over 10 electrodes on the scalp at 300–500 ms (A–C) and 500–800 ms (D–F) after sleep. The LPP amplitude in 300–500 ms was larger in the odor condition than in the control condition. After odor stimulation during REM sleep, the LPP amplitude in 500–800 ms increased only for old negative images. Tukey error bars indicate 1.5 times the 25th and 75th percentiles or maximum and minimum values. *:*p *< .05 with Bonferroni correction.

The main effect of the condition was obtained for the emotional picture task after sleep at 500–800 ms (*F*(1, 1800) = 5.15, *p* = .023), which demonstrated that the amplitude of the LPP in the odor condition (2.13 ± 2.04 μV) was larger than that in the control condition (1.81 ± 1.74 μV) (Supplementary Table S4). A three-way interaction of the condition, image type, and image novelty was also observed (*F*(2, 1800) = 3.74, *p* = .02; Supplementary Table S4). Since the effect of the electrode was not included in the interaction, data from 10 electrodes were combined to perform a post-hoc test for the condition, image novelty, and image type (Figure 6). Multiple comparisons revealed that in old images, the amplitude of the LPP relative to negative images in the odor condition was larger than in the control condition (*p* = .002 for uncorrected; *p* = .012 for Bonferroni corrected, Figure 6D). However, no effect was observed on the amplitude of the LPP for neutral (*p* = .356 for uncorrected; *p* = 1.000 for Bonferroni corrected, Figure 6E) and positive images (*p* = .980 for uncorrected; *p *= 1.000 for Bonferroni corrected, Figure 6F) between the conditions. Moreover, in the new image, no effect was observed on the LPP amplitude for the negative (*p* = 0.683 for uncorrected; *p* = 1.000 for Bonferroni corrected, Figure 6D), neutral (*p* = .969 for uncorrected; *p* = 1.000 for Bonferroni corrected, Figure 6E) and positive images (*p* = .051 for uncorrected; *p* = .308 for Bonferroni corrected Figure 6F) between the conditions. Thus, after olfactory stimulation during REM sleep, the LPP amplitude increased only from 500 to 800 ms for the negative image (old negative image) viewed before sleep. After excluding outliers for each combination of Type, Novelty, and electrode (12 data points) and conducting the same analysis, the main effect of the condition remained (*F*(1, 1788) = 6.72, *p* = .0098). In contrast, the three-way interaction among condition, image type, and image novelty was not statistically significant (*F*(2, 1788) = 2.74, *p* = .065). The robustness of these results should be verified by increasing the sample size.

**Figure 7.**
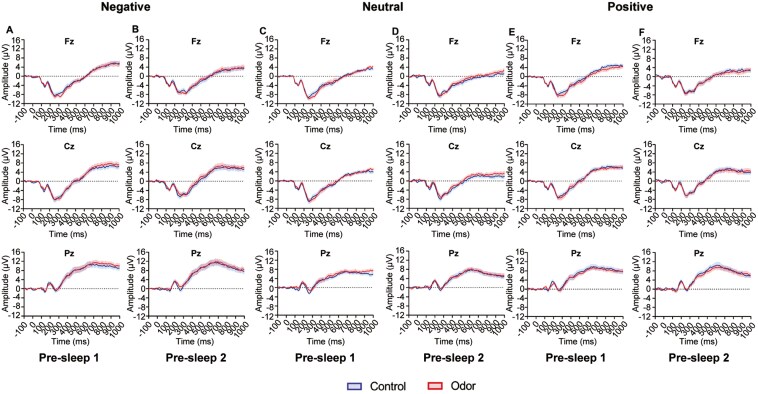
Event-related potentials elicited by negative (A, B), neutral (C, D), and positive (E, F) images during the pre-sleep 1 (A, C, E) and pre-sleep 2 (B, D, F) sessions. Red lines indicate the odor stimulation condition, and blue lines indicate the control condition. Shaded areas represent the standard error of the mean.

Importantly, no main effect of condition nor interaction of condition was observed before sleep for 300–500 ms and 500–800 ms (pre-sleep 1: Supplementary Table S2; pre-sleep 2: Supplementary Table S3). The LPP amplitude before sleep was similar between 300–500 ms and 500–800 ms in the odor and control conditions (Figure 7).

### Memory accuracy during the emotional picture task

**Figure 8.**
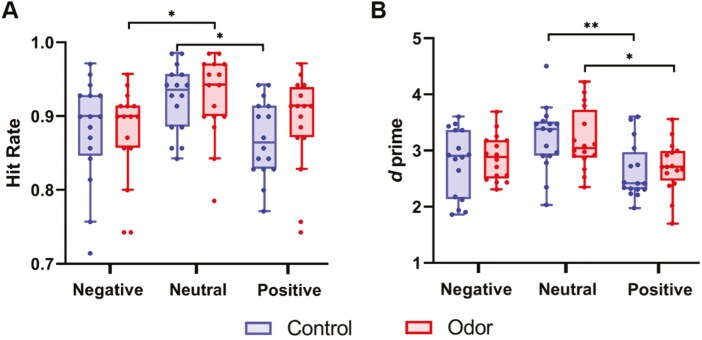
The influence of odor condition on the memories. (A) Hit rate. (B) *d’*. There were no differences in memory accuracy between conditions. Red boxes represent the odor condition, and blue boxes represent the control condition. Tukey error bars indicate 1.5 times the 25th and 75th percentiles or maximum and minimum values. *:*p *< .05 with Bonferroni correction, **:*p *< .01 with Bonferroni correction.

Figure 8 shows the results of memory accuracy during the task after sleep. A linear mixed model revealed a main effect of the image type (*F*(2,90) = 7.56, *p* = .0009) based on the hit rate (Figure 8A). Surprisingly, after multiple comparisons, a significantly better performance was found on the neutral images compared to negative images (*p* = .0013 for uncorrected; *p* = .0038 for Bonferroni corrected) and on the neutral images compared to positive images (*p* = .001 for uncorrected; *p* = .003 for Bonferroni corrected). No main effect of condition (*F*(1, 90) = 0.47, *p* = .49) or interaction was observed between the condition and image type (*F*(2, 90) = 0.60, *p* = .55). Results of *d’* (Figure 8B) also demonstrated a main effect of image type (*F*(2, 90) = 9.13, *p* = .0002), but no main effect of condition (*F*(1, 90) = 0.22, *p* = .64) or interaction between the condition and image type (*F*(2, 90) = 0.17, *p* = .85). Multiple comparisons revealed better performances on the neutral images than positive images (*p* ≤ .0001 for uncorrected; *p* = .0002 for Bonferroni corrected) and negative images (*p* = .0046 for uncorrected; *p* = .0137 for Bonferroni corrected). Reanalysis was conducted after excluding outliers for HR and *d’* (HR: 3 data points from negative images; *d’*: 1 data point from neutral images). For the hit rate, the results remained unchanged after excluding outliers. For *d’*, no change was observed between the positive and neutral images; however, the difference between the negative and neutral images was no longer significant after multiple comparison corrections (*p* = .03 uncorrected; *p* = .09 Bonferroni corrected).

## Discussion

Our objective was to investigate whether REM sleep facilitates the consolidation of emotional memory and reduces emotional intensity through the use of TMR. The results demonstrated that contrary to the hypothesis, no significant difference was observed in memory accuracy between the two conditions, and the LPP amplitude increased for the old negative image following olfactory stimuli presentation during REM sleep in the 500–800-ms period. The LPP amplitude for 300–500 ms and 500–800 ms after sleep was larger in the odor condition than in the control condition. Additionally, while no effect of olfactory stimulation for arousal was found immediately after viewing the image, the results of the profile of the mood states revealed that olfactory stimulation during REM sleep reduced negative mood the following day. In contrast, when multiple comparison correction was not applied or when analyses were conducted after excluding outliers, no clear TMR-specific effects on LPP and negative mood were observed other than the increase in LPP amplitude in the positive direction in the odor condition. As these results may reflect unstable outcomes due to the small sample size, further investigation is warranted.

Initially, we hypothesized that TMR during REM sleep would reduce the amplitude of the LPP. However, contrary to our expectations, our pilot study observed an increased LPP amplitude upon awakening, but only in response to unpleasant emotional image stimuli previously encountered when TMR was applied. Previous research has reported inconsistent effects of sleep on LPP amplitude. For instance, after REM-rich sleep, the LPP amplitude elicited by emotional images experienced before sleep, in the 300–500 ms range, was found to increase compared to that for novel unpleasant images [13], a finding that aligns with our results. Conversely, the maximum amplitude in the 600–1000 ms range was reported to decrease 10 hours post-sleep compared to post-wakefulness [48], a result that diverges from our findings. However, these discrepancies may be attributed to variations between groups included in each study, such as differences in circadian phase and sleep homeostasis. Given that LPP amplitude is affected by circadian rhythms and sleep deprivation [49, 50], our study resolved these issues through TMR. Because the robustness of the results could not be confirmed due to the small sample size, further investigation is warranted. Additionally, the control condition in this study involved an odorless stimulus. Therefore, whether the enhancement of LPP was due to memory reprocessing through TMR or simply the presentation of an odor stimulus during REM sleep remains unclear. Furthermore, since the stimulus intervention was applied only during REM sleep, it is also unclear whether the observed effects are specific to REM sleep.

The specific type of emotional processing facilitated, however, remains unclear. While the debate on LPP’s function persists, LPP is akin to the P3 typically observed in oddball tasks and is thought to reflect the significance of stimuli [51] or the operation of constructing long-term memory models [52]. It is conceivable that REM sleep preferentially enhances the significance of unpleasant stimuli or the construction of long-term emotional memory models. Based on this discussion, it is difficult to conclude whether the enhancement of LPP amplitude in this study reflects an increase of emotional charge or the facilitation of memory consolidation.

Our preliminary results also demonstrated that olfactory stimulation during REM sleep decreased negative mood, as evidenced by decreased tension, anxiety, and total mood disturbance the following day. Hence, changes of the processing in response to negative emotional stimuli was accompanied with the reduction of negative mood. However, total mood disturbance in the control condition also significantly decreased when multiple comparison correction was not applied. Therefore, it is not possible to conclude from the present study whether the effects were specifically due to TMR during REM sleep. Moreover, in this study, we focused on changes in subjective mood before and after sleep, rather than as a reaction to the stimulus itself. Therefore, it is important to note that the current design does not allow us to determine whether the reduction in negative mood under the TMR condition is due to the reprocessing effect during REM sleep or the effect of olfactory stimuli presented during REM sleep itself. Further investigation is needed to clarify this distinction by incorporating a condition with no olfactory stimulus before sleep and comparing scenarios where olfactory stimuli are applied before waking and during REM sleep to those where stimuli are only presented during REM sleep.

An unexpected finding was that in the odor condition, the LPP amplitudes tended to shift to a more positive direction compared to the control condition in both the 300–500 ms and 500–800 ms time windows. This result remained robust even after excluding outliers. For the 300–500 ms amplitude, no interaction with novelty was observed. If the increased positive amplitude in the odor condition occurred independent of the novelty, the effect could be attributable to the odor stimulation itself rather than memory reactivation. However, the underlying phenomenon cannot be determined from the present study, and further research is warranted.

Previous TMR studies employed negative and neutral images in their experiments [18, 19]. In contrast, in our pilot study, neutral, negative, and positive images were utilized to determine the preferential processing of each image type. The preliminary findings revealed that TMR during REM sleep enhanced the amplitude of the LPP only for negative images viewed before sleep, while it did not impact the LPP for neutral and positive images. This effect may be due to sleep selectively processing memories of highly salient negative images. The selectivity of sleep-dependent memory processing is influenced by the salience of information during encoding [53], with highly salient information more likely to be preferentially processed during sleep. Since subjective arousal was significantly higher for negative images than positive images before sleep in this study, negative images with higher arousal were likely preferentially processed during sleep. In addition, negative and positive emotional processes possibly did not occur simultaneously. Research has demonstrated that cells corresponding to positive and negative behaviors within different areas of the basolateral amygdala inhibit each other’s neuronal activation [54]. Thus, the salient negative image in this study may have been preferentially processed during REM sleep and suppressed memory consolidation for the positive image, which in turn was reflected in the increased LPP amplitude only for the old negative images following sleep.

Our pilot study did not demonstrate any effect of TMR on arousal after viewing emotional images. Two previous studies applying TMR during REM sleep have shown inconsistent results in assessing arousal levels in response to images; one found no effect of TMR [19], while the other reported a reduction in arousal ratings for images with TMR during REM sleep [18]. Regarding this inconsistency, Hutchison et al. [18] highlighted a key difference in the timing of arousal ratings between the two studies. In Lehmann et al.‘s study [19], the learning task was conducted after the arousal rating, whereas in Hutchison et al.’s study [18], the arousal rating occurred after the learning phase. Based on this difference, Hutchison et al. [18] proposed that habituation from the learning and retrieval tasks, which followed the arousal rating task in the pre-sleep session, may have occurred in the study by Lehmann et al. [19] However, in line with Hutchison et al. [18], our study compared arousal immediately before and after sleep, using the first pre-sleep session as a baseline. Despite this, we still found that TMR during REM did not affect the arousal levels of the images. One possible explanation for this could be the differences in the stimuli used for TMR (auditory vs. olfactory stimuli), showing that olfactory-based TMR may have a weaker effect than auditory-based TMR, contrary to the assumptions we made. Although a meta-analysis of TMR showed that the effect sizes of auditory and olfactory stimuli are comparable [17], the differences in the effects of auditory and olfactory stimuli on TMR, limited to REM sleep, remain unclear. Further investigation is needed to elucidate the reason for this discrepancy. Another reason may be the issue of insufficient statistical power. Given the exploratory nature of the present study, future research should include a larger number of participants to validate the findings.

In this study, behavioral assessments did not reveal significant differences in memory accuracy between conditions. This preliminary finding aligns with existing literature [18, 19], which showed that TMR during REM sleep does not improve memory performance for emotional memories after sleep. A recent study using TMR has suggested that REM sleep may instead be involved in forgetting emotional memories [55]. In contrast, TMR during NREM sleep or slow-wave sleep has been reported to facilitate the consolidation of emotional stimuli [19, 55, 56], although such effects are not consistently observed [18, 57]. As this study did not apply TMR during NREM sleep, we cannot draw conclusions about the distinct roles of different sleep stages in emotional memory consolidation, highlighting the need for further investigation in this area. In addition, the high hit rates (Negative images: 87.9 ± 6.7%; Neutral images: 92.5 ± 5.0%; Positive images: 88.3 ± 5.9%) resulting from conducting the memory recognition task after a relatively short 12-hour interval may have produced a ceiling effect, making it difficult to detect meaningful differences.

Our study has some limitations. First, we cannot exclude the possibility that olfactory stimulation during REM sleep may have effects unrelated to TMR. It would be beneficial to include a condition where olfactory stimuli are not presented during the emotional task but are introduced during REM sleep, to better confirm the effects of TMR upon awakening. Second, to evaluate the emotional responses based on the Sleep to Forget, Sleep to Remember hypothesis from a multidimensional perspective, it is necessary to consider the measures of the autonomic nervous system, such as heart rate variability. Third, this study did not account for the timing of inhalation when delivering the olfactory stimulation. Breath-synchronized olfactory stimulators could enhance the effect of TMR on subjective measures. As employed in previous research [58], synchronizing TMR with breathing could yield a stronger effect. Fourth, the memory task design should be considered to determine the impact of TMR on behavioral measures of memory accuracy. A previous review [59] mentioned that incorporating free recall tasks alongside recognition tasks, and considering the baseline results of the recognition task, enhances the likelihood of observing the effects of sleep on memory. Furthermore, the recognition task conducted after 12 hours may have exhibited a ceiling effect, making it difficult to detect differences between the two conditions. Upon considering the task employed and the study design in future research, the effects of TMR during REM sleep could also be demonstrated in behavioral measures for memory accuracy. Fifth, as described above, based on the view that the LPP reflects either the significance of stimuli [51] or the operation of constructing long-term memory models [52], it is difficult to draw a conclusion as to whether the present results reflect the emotional or memory-related aspects of the Sleep to Forget, Sleep to Remember hypothesis. Additionally, further research is warranted to examine the effects of TMR on LPP in the absence of a memory recognition task. In this study, the memory recognition task, which enquired whether the participant had seen the emotional image before sleep, was performed after waking. Implementation of the memory recognition task may have induced stronger attention and increased the LPP amplitude when the old image was presented. However, LPP amplitude reportedly decreases with repeated image presentation when the task does not include a memory recognition task [60]. Therefore, future research should investigate changes in LPP amplitude without involving memory tasks to determine whether TMR during REM sleep facilitates habituation to both negative and positive images. Sixth, it is conceivable that conducting TMR employed in this experiment over multiple nights may lead to a reduction in arousal levels in response to images. Indeed, studies have shown that the effects of sleep on distress related to trauma films become apparent after the third day [61], and the impacts on heart rate deceleration and valence are more pronounced 1 week after encoding rather than just 10 hours later [48]. Additionally, another study has demonstrated that the facilitative effects on rule abstraction appear 1 week after TMR during REM sleep [62]. Future research should, therefore, explore the long-term effects of TMR during REM sleep. Finally, the relatively small number of participants limits the ability to draw definitive conclusions. A previous meta-analysis study showed that the effect size of TMR during non-REM sleep is estimated to be small to moderate, while TMR during REM sleep did not show significant effectiveness [17]. To confirm the reproducibility of these findings, future studies with larger sample sizes will be required.

In conclusion, this pilot study with preliminary findings demonstrated that olfactory stimulation during REM sleep which was accompanied with the emotional memory task before sleep heightened the LPP amplitude in response to negative images viewed before bedtime. This finding indicates that REM sleep plays an important role in selectively changes response to negative stimuli encountered in the past. Additionally, a reduction in negative mood was observed after awakening especially in the TMR condition. However, no significant differences were observed in emotional memory consolidation and subjective evaluation of the emotional pictures between conditions in the post-sleep task. From our pilot study, whether the observed effects were due to the reprocessing of unpleasant stimuli during REM sleep or to olfactory stimulation itself during REM sleep remains unclear. If reprocessing during REM sleep contributes to the increase in the LPP amplitude in response to unpleasant old stimuli, then pairing with an odor before sleep, compared to no pairing, would result in an increased LPP amplitude specifically to old negative images when the same odor is presented during REM sleep. Moreover, it is still unknown whether these effects are specific to REM sleep. If the increase in the LPP to negative old images after waking, resulting from the reprocessing of previously encountered negative images, occurs specifically during REM sleep, then TMR during REM sleep would lead to a greater increase in the LPP to old negative images compared to TMR conducted during NREM sleep or wakefulness. By increasing the sample size and adding conditions, replication and extension of these preliminary findings are expected to advance our understanding of the role of REM sleep in emotional processing.

## Supplementary Material

zpaf034_suppl_Supplementary_Tables_S1-S4_Figures_S1-S4

## Data Availability

The data underlying this article will be shared upon reasonable request to the corresponding author.
