# Supplementary material for: Targeted memory reactivation during REM sleep may selectively enhance the late positive potential amplitude in previously encountered negative images: preliminary findings
Source: Sleep Adv. 2025 May 24;6(2):zpaf034. doi: 10.1093/sleepadvances/zpaf034 (PMC12199615; doi:10.1093/sleepadvances/zpaf034)
Supplement: zpaf034_suppl_Supplementary_Tables_S1-S4_Figures_S1-S4 [file zpaf034_suppl_supplementary_tables_s1-s4_figures_s1-s4.docx]

# Supplemental Materials

# Targeted memory reactivation during REM sleep may selectively enhance the late positive potential amplitude in previously encountered negative images: Preliminary findings

Kazuki Sato^1,2^, Satomi Okabe^1,2,3,4^, Yoko Suzuki^2^, Takashi Abe^2^

^1^ Graduate School of Comprehensive Human Science, University of Tsukuba

^2^ International Institute for Integrative Sleep Medicine (WPI-IIIS), University of Tsukuba

^3^ Department of Applied Biological Chemistry, Graduate School of Agricultural and Life Sciences, The University of Tokyo

^4^ Japan Society for Promotion of Science

Corresponding author:

Takashi Abe, Ph.D., International Institute for Integrative Sleep Medicine (WPI-IIIS), University of Tsukuba, 1-1-1 Tennodai, Tsukuba, Ibaraki 305-8575, Japan

E-mail: abe.takashi.gp@u.tsukuba.ac.jp

**Figure S1.** Profile of Mood States 2nd Edition-Adult Short items for (**A**) Anger-Hostility (AH), (**B**) Confusion-Bewilderment (CB), (**C**) Friendliness (F), and (**D**) Vigor-Activity (VA)
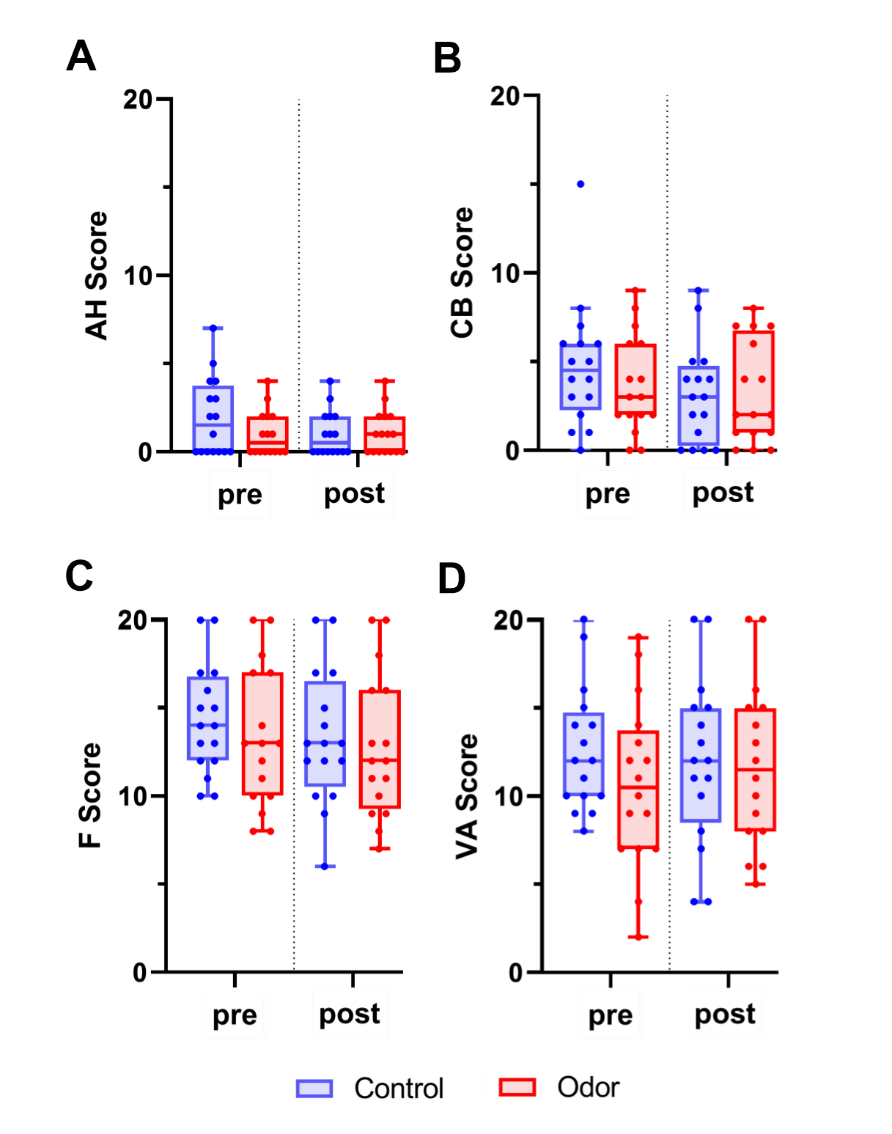


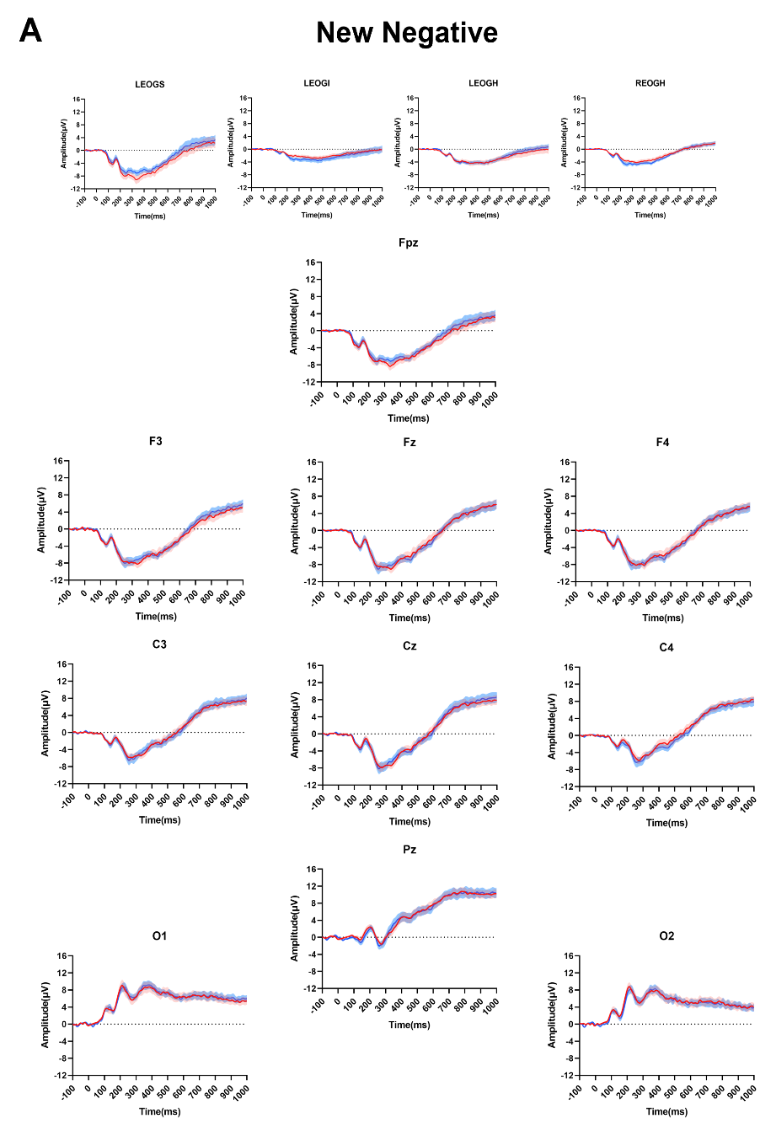

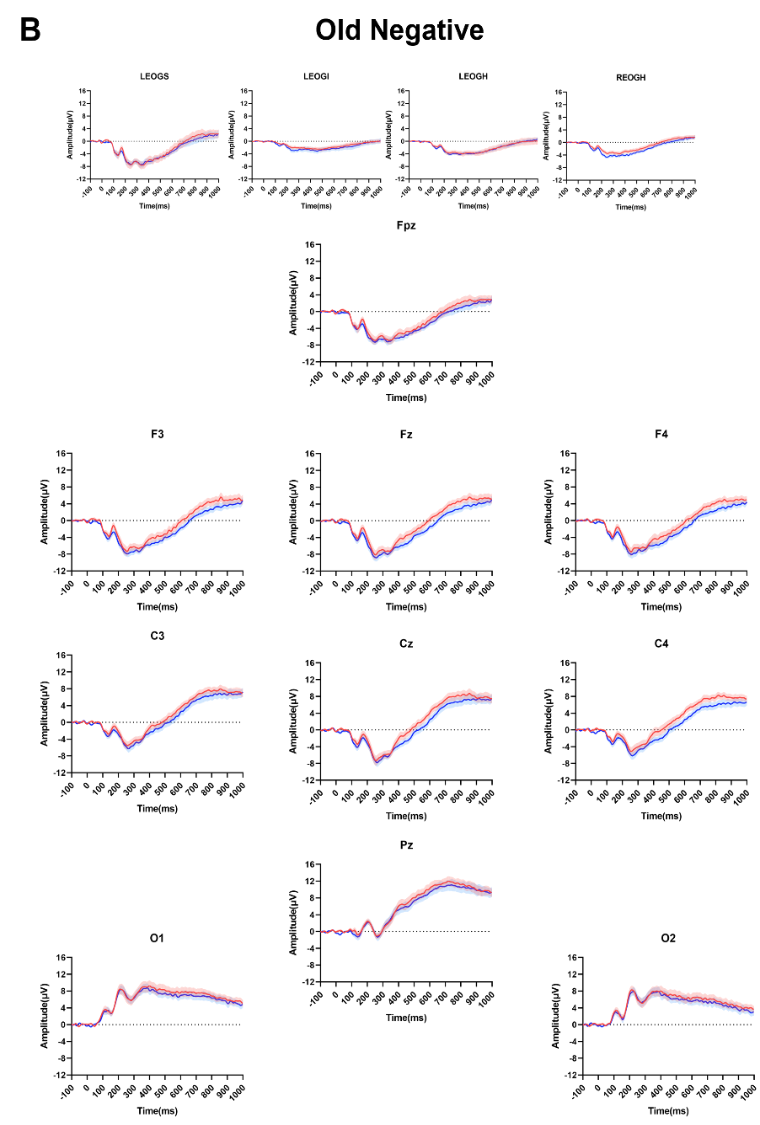

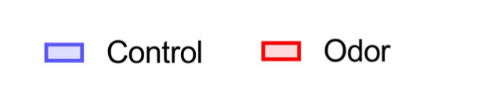


**Figure S2**. Event-related potentials on the scalp and electrooculogram (EOG) channel of the new (**A**) and old (**B**) negative images after sleep. Red lines represent the odor stimulation, and blue lines represent the control conditions. The shaded area corresponds to the standard error of the mean.


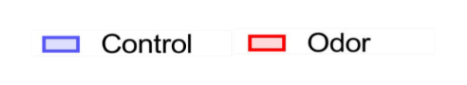

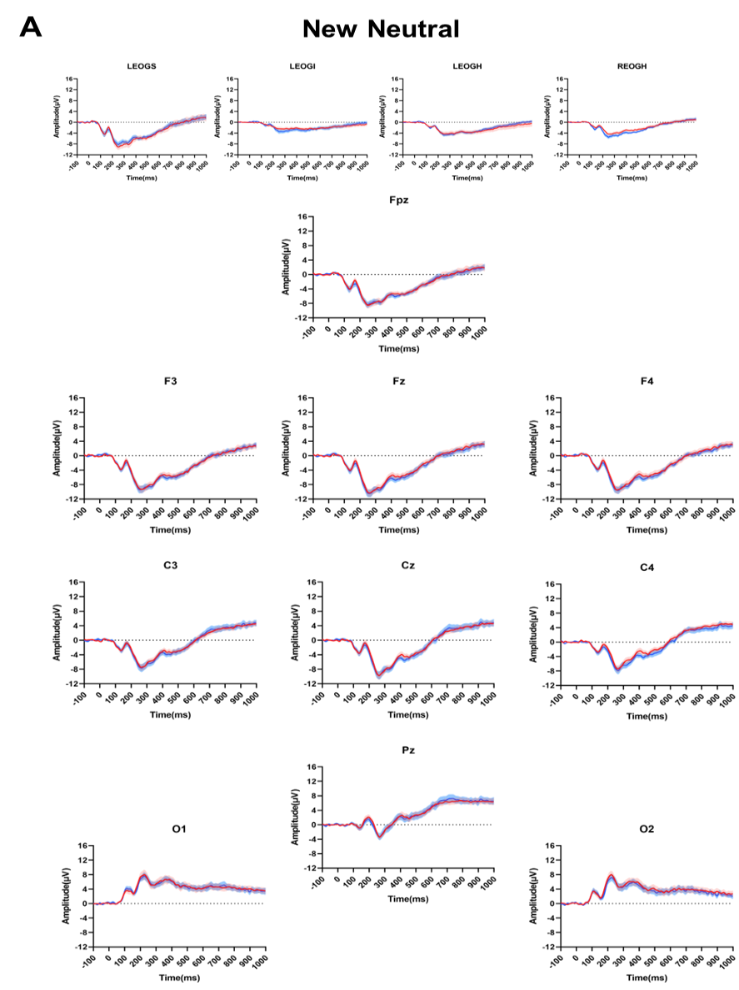

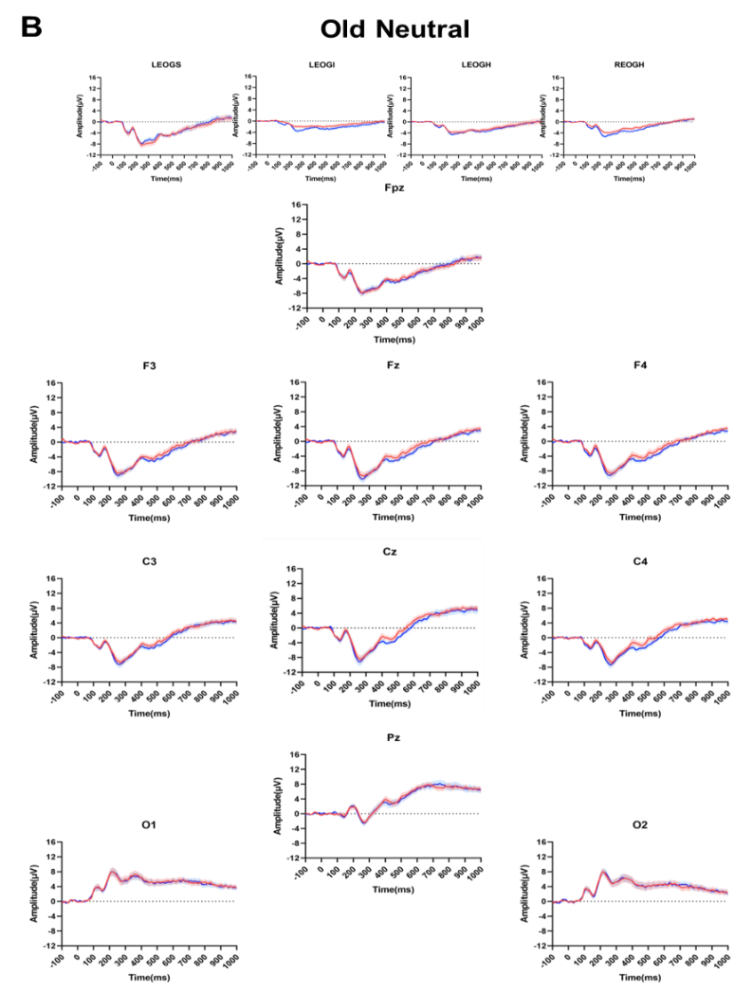


**Figure S3**. Event-related potentials on the scalp and electrooculogram (EOG) channel of the new (**A**) and old (**B**) neutral images after sleep. Red lines represent odor stimulation, and blue lines represent control conditions. The shaded area corresponds to the standard error of the mean.


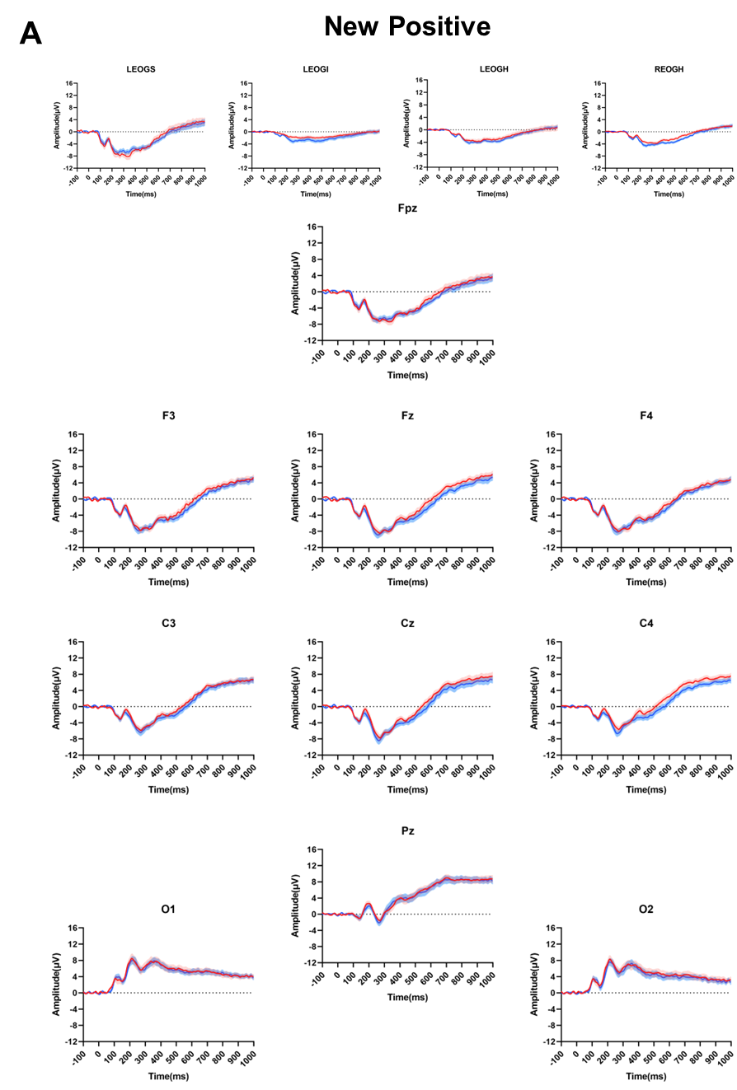

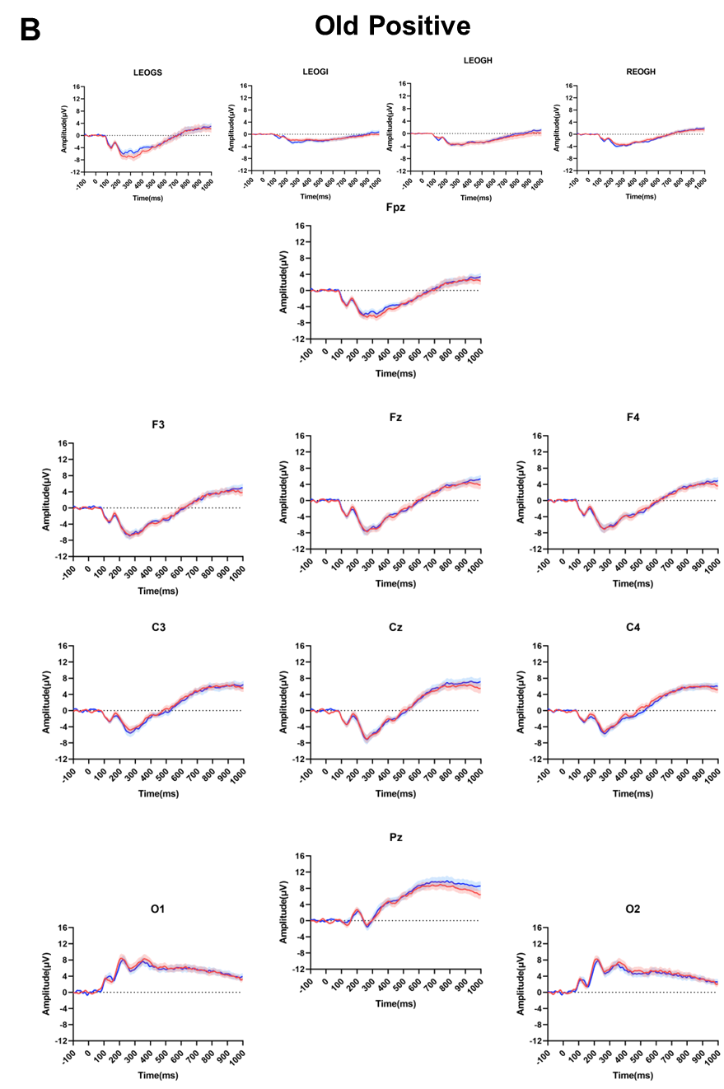

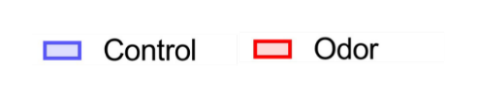


**Figure S4**. Event-related potentials on scalp and electrooculogram (EOG) channel of the new (**A**) and old (**B**) positive images after sleep. Red lines represent the odor stimulation, and blue lines represent the control conditions. The shaded area corresponds to the standard error of the mean.

**Table S1.** Summary of the International Affective Picture System and Open Affective Standardized Image Set rating for each set (mean±SD)

| Emotion | Dimensions | Set 1 | Set 2 | Set 3 | Set 4 | *F(3,276)* | *p* |
| --- | --- | --- | --- | --- | --- | --- | --- |
| Negative | Valence | 2.47±0.68 | 2.40±0.61 | 2.42±0.60 | 2.35±0.57 | 0.43 | 0.73 |
|  | Arousal | 5.79±0.77 | 5.75±0.71 | 5.77±0.75 | 5.78±0.74 | 0.04 | 0.99 |
| Neutral | Valence | 5.06±0.49 | 5.14±0.39 | 5.18±0.43 | 5.13±0.44 | 0.89 | 0.45 |
|  | Arousal | 3.32±0.48 | 3.30±0.45 | 3.27±0.50 | 3.34±0.47 | 0.34 | 0.80 |
| Positive | Valence | 6.94±0.65 | 6.86±0.66 | 6.84±0.63 | 6.84±0.60 | 0.44 | 0.72 |
|  | Arousal | 5.30±0.93 | 5.21±0.79 | 5.27±0.81 | 5.17±0.78 | 0.34 | 0.80 |

**Table S2.** Results of the statistical analyses of the event-related potential before sleep (Pre-sleep 1) obtained using a linear mixed model

| Effect | *df1* | *df2* | 300-500ms | | 500-800ms | |
| --- | --- | --- | --- | --- | --- | --- |
|  |  |  | *F* | *P* | *F* | *P* |
| Condition | 1 | 900 | 0.31 | 0.58 | 0.04 | 0.84 |
| Channel | 9 | 900 | 150.32 | <0.0001 | 91.38 | <0.0001 |
| Image type | 2 | 900 | 4.81 | 0.01 | 38.91 | <0.0001 |
| Condition⋅Channel | 9 | 900 | 0.22 | 0.99 | 0.31 | 0.97 |
| Condition⋅Image type | 2 | 900 | 0.01 | 0.99 | 0.77 | 0.46 |
| Image type⋅Channel | 18 | 900 | 0.32 | 1.00 | 0.50 | 0.96 |
| Condition⋅Image type⋅Channel | 18 | 900 | 0.02 | 1.00 | 0.03 | 1.00 |

**Table S3.** Results of the statistical analyses of the event-related potential before sleep (Pre-sleep 2) obtained using a linear mixed model

| Effect | *df1* | *df2* | 300–500ms | | 500–800ms | |
| --- | --- | --- | --- | --- | --- | --- |
|  |  |  | *F* | *P* | *F* | *P* |
| Condition | 1 | 900 | 1.30 | 0.26 | 1.42 | 0.23 |
| Channel | 9 | 900 | 147.06 | <0.0001 | 79.95 | <0.0001 |
| Image type | 2 | 900 | 3.69 | 0.025 | 25.23 | <0.0001 |
| Condition⋅Channel | 9 | 900 | 0.09 | 1.00 | 0.08 | 1.00 |
| Condition⋅Image type | 2 | 900 | 0.04 | 0.96 | 1.15 | 0.32 |
| Image type⋅Channel | 18 | 900 | 0.39 | 0.99 | 0.47 | 0.97 |
| Condition⋅Image type⋅Channel | 18 | 900 | 0.03 | 1.00 | 0.05 | 1.00 |

**Table S4.** Results of the statistical analyses of the event-related potential after sleep (Post-sleep) obtained using a linear mixed model

| Effect | *df1* | *df2* | 300–500ms | | 500–800ms | |
| --- | --- | --- | --- | --- | --- | --- |
|  |  |  | *F* | *p* | *F* | *p* |
| Condition | 1 | 1800 | 4.05 | 0.044 | 5.15 | 0.023 |
| Channel | 9 | 1800 | 380.23 | <0.0001 | 160.44 | <0.0001 |
| Novelty of image | 1 | 1800 | 49.51 | <0.0001 | 27.27 | <0.0001 |
| Image type | 2 | 1800 | 18.94 | <0.0001 | 56.29 | <0.0001 |
| Condition⋅Channel | 9 | 1800 | 0.42 | 0.93 | 0.26 | 0.99 |
| Condition⋅Novelty of image | 1 | 1800 | 0.56 | 0.45 | 0.96 | 0.33 |
| Condition⋅Image type | 2 | 1800 | 0.42 | 0.66 | 0.37 | 0.69 |
| Novelty of image⋅Channel | 9 | 1800 | 1.51 | 0.14 | 0.33 | 0.96 |
| Channel⋅Image type | 18 | 1800 | 1.65 | 0.041 | 1.49 | 0.084 |
| Novelty of image⋅Image type | 2 | 1800 | 0.02 | 0.98 | 0.49 | 0.62 |
| Channel⋅Novelty of image⋅Image type | 18 | 1800 | 0.02 | 1.00 | 0.03 | 1.00 |
| Condition⋅Novelty of image⋅Image type | 2 | 1800 | 1.58 | 0.21 | 3.74 | 0.02 |
| Condition⋅Channel⋅Novelty of image | 9 | 1800 | 0.01 | 1.00 | 0.04 | 1.00 |
| Condition⋅Channel⋅Image type | 18 | 1800 | 0.05 | 1.00 | 0.03 | 1.00 |
| Condition⋅Channel⋅Novelty of image⋅Image type | 18 | 1800 | 0.07 | 1.00 | 0.09 | 1.00 |

**International Affective Picture System picture numbers for the images used in this study**

Set 1

Negative: 1111, 1120, 1200, 1271, 1300, 1301, 1525, 1931, 2095, 2120, 2751, 2900, 2981, 3001, 3015, 3016, 3019, 3030, 3051, 3068, 3101, 3102, 3110, 3120, 3140, 3168, 3261, 3301, 3400, 6190, 6200, 6231, 6244, 6250, 6263, 6300, 6312, 6313, 6314, 6315, 6360, 6415, 6510, 6520, 6561, 6563, 6570, 6821, 7380, 9140, 9181, 9302, 9419, 9425, 9452

Neutral: 2036, 2102, 2104, 2190, 2191, 2200, 2206, 2210, 2215, 2235, 2270, 2271, 2273, 2280, 2305, 2351, 2383, 2384, 2393, 2394, 2397, 2400, 2441, 2480, 2493, 2512, 2518, 2525, 2570, 2593, 2595, 2745.1, 2791, 5390, 5471, 1616, 2100, 7002, 7004, 7006, 7096, 7012, 7014, 7016, 7025, 7034, 7035, 7036, 7037, 7040, 7041, 7090, 7550, 7493, 7590

Positive: 1440, 1463, 1710, 1999, 2030, 2154, 2300, 2303, 2340, 2352, 2398, 2530, 4608, 4650, 4658, 4660, 4676, 4680, 4687, 4689, 4690, 4694, 4695, 5470, 5611, 5623, 5660, 5725, 5781, 5830, 5836, 7220, 7230, 7350, 7405, 7451, 7460, 7480, 7488, 7660, 8030, 8031, 8120, 8180, 8185, 8186, 8190, 8200, 8210, 8300, 8350, 8380, 8400, 8461, 8490

Set 2

Negative: 1050, 1220, 1304, 2691, 2710, 3000, 3053, 3061, 3064, 3100, 3131, 3180, 3191, 3212, 3215, 3225, 3350, 3530, 5971, 6022, 6213, 6243, 6338, 6410, 6550, 6571, 6831, 7359, 8485, 9040, 9050, 9184, 9250, 9254, 9325, 9340, 9405, 9412, 9420, 9424, 9428, 9433, 9491, 9520, 9570, 9571, 9599, 9611, 9622, 9635.1, 9901, 9904, 9909, 9920, 9930

Neutral: 5731, 2020, 7000, 2221, 2308, 2377, 2385, 2411, 2445, 2485, 2491, 2499, 2514, 2580, 2720, 2830, 2870, 2980, 4500, 4573, 5120, 5510, 5531, 5534, 7001, 7011, 7018, 7026, 7032, 7039, 7042, 7045, 7055, 7059, 7062, 7010, 7100, 7140, 7170, 7183, 7186, 7192, 7207, 7224, 7235, 7242, 7283, 7290, 7495, 7500, 7175, 7595, 7920, 8312, 9210

Positive: 1340, 1659, 1721, 2040, 2070, 2080, 2155, 2165, 2216, 2345, 2362, 2550, 2900.2, 4220, 4311, 4597, 4600, 4606, 4610, 4614, 4623, 4626, 4641, 4644, 4651, 4652, 4669, 4670, 4800, 5210, 5301, 5480, 5629, 5820, 5833, 5910, 7270, 7279, 7390, 7430, 7492, 7502, 7580, 8021, 8040, 8080, 8116, 8130, 8162, 8179, 8206, 8371, 8500, 8531, 9156

Set 3

Negative: 1274, 2053, 2683, 2692, 2717, 3010, 3059, 3062, 3069, 3080, 3103, 3150, 3170, 3195, 3216, 3230, 3550, 6020, 6210, 6220, 6260, 6350, 6530, 6555, 6560, 6825, 6834, 7135, 9006, 9042, 9075, 9160, 9183, 9185, 9252, 9295, 9322, 9326, 9409, 9413, 9426, 9429, 9495, 9530, 9590, 9600, 9620, 9623, 9800, 9900, 9902, 9905, 9910, 9921, 9940

Neutral: 1670, 2026, 2107, 2272, 2357, 2381, 2390, 2435, 2446, 2488, 2506, 2516, 2620, 2749, 2840, 2880, 4000, 4510, 4605, 5130, 5520, 5532, 5740, 7003, 7013, 7019, 7030, 7033, 7043, 7050, 7053, 7057, 7060, 7080, 7110, 7150, 7160, 7179, 7184, 7187, 7205, 7233, 7236, 7249, 7285, 7300, 7490, 7512, 7546, 7700, 7705, 7950, 8465, 9260, 9700

Positive: 1540, 1660, 1722, 2045, 2057, 2071, 2091, 2158, 2208, 2224, 2346, 2389, 4150, 4290, 4601, 4607, 4611, 4617, 4624, 4628, 4643, 4653, 4656, 4677, 4810, 5215, 5450, 5600, 5626, 5700, 5825, 5849, 5982, 7250, 7282, 7330, 7400, 7481, 7499, 7650, 8033, 8041, 8158, 8163, 8178, 8208, 8340, 8370, 8420, 8470, 8492, 8496, 8502, 8510, 8540

Set 4

Negative: 1202, 2688, 2703, 2730, 2800, 2811, 3017, 3060, 3063, 3071, 3130, 3160, 3185, 3213, 3220, 3266, 3500, 4664.2, 6021, 6212, 6230, 6242, 6370, 6540, 6830, 6836, 8230, 9043, 9120, 9163, 9187, 9253, 9300, 9301, 9321, 9332, 9400, 9410, 9414, 9423, 9427, 9430, 9500, 9560, 9592, 9610, 9621, 9630, 9810, 9903, 9908, 9911, 9922, 9925, 9941

Neutral: 2002, 2038, 2214, 2279, 2359, 2382, 2396, 2440, 2484, 2489, 2495, 2513, 2579, 2702, 2850, 2890, 4233, 4571, 5040, 5500, 5530, 5533, 6150, 7009, 7017, 7020, 7031, 7038, 7044, 7052, 7056, 7058, 7061, 7081, 7130, 7161, 7165, 7180, 7185, 7190, 7217, 7234, 7237, 7255, 7287, 7354, 7491, 7513, 7547, 7710, 8311, 9002, 9070, 9360, 9401

Positive: 1590, 1650, 1720, 1731, 2050, 2058, 2075, 2150, 2160, 2209, 2344, 2347, 2391, 2655, 4250, 4525, 4575, 4599, 4603, 4609, 4612, 4619, 4640, 4645, 4659, 4668, 4681, 4698, 5199, 5260, 5270, 5460, 5621, 5628, 5814, 5829, 5890, 5994, 7200, 7260, 7410, 7470, 7501, 7508, 7570, 8001, 8034, 8090, 8161, 8170, 8193, 8220, 8499, 8501, 8503

**Open Affective Standardized Image Set picture numbers for the images used in this study**

Set 1

Negative: I24, I118, I150, I234, I276, I302, I306, I322, I327, I440, I518, I794, I850, I865, I889

Neutral: I29, I48, I80, I113, I141, I179, I206, I289, I389, I393, I427, I504, I623, I716, I786

Positive: I30, I59, I134, I188, I255, I264, I270, I334, I338, I372, I377, I469, I510, I660, I874

Set 2

Negative: I26, I120, I208, I248, I282, I303, I307, I323, I328, I451, I641, I729, I825, I851, I867

Neutral: I31, I67, I98, I138, I158, I187, I218, I353, I390, I396, I432, I515, I629, I718, I796

Positive: I32, I60, I143, I189, I256, I268, I308, I335, I339, I459, I463, I470, I511, I661, I876

Set 3

Negative: I27, I122, I209, I273, I284, I304, I317, I324, I333, I437, I452, I714, I828, I857, I869

Neutral: I75, I72, I99, I139, I175, I199, I221, I354, I391, I499, I488, I524, I686, I723, I805

Positive: I34, I65, I159, I190, I262, I269, I291, I336, I340, I466, I486, I505, I516, I811, I878

Set 4

Negative: I82, I136, I210, I274, I283, I287, I319, I320, I326, I439, I496, I769, I848, I864, I871

Neutral: I44, I42, I105, I140, I177, I200, I224, I358, I395, I426, I397, I525, I706, I747, I822

Positive: I35, I132, I186, I252, I263, I271, I293, I337, I352, I468, I487, I508, I616, I813, I882
